# The Israel National Sera Bank: Methods, Representativeness, and Challenges

**DOI:** 10.3390/ijerph18052280

**Published:** 2021-02-25

**Authors:** Ravit Bassal, Dani Cohen, Manfred S. Green, Lital Keinan-Boker

**Affiliations:** 1 Israel Center for Disease Control, Ministry of Health, Sheba Medical Center, Ramat Gan 5262160, Israel; Lital.Keinan2@MOH.GOV.IL; 2 Department of Epidemiology and Preventive Medicine, School of Public Health, Sackler Faculty of Medicine, Tel Aviv University, Tel Aviv 6997801, Israel; dancohen@tauex.tau.ac.il; 3 School of Public Health, University of Haifa, Haifa 3498838, Israel; manfred.s.green@gmail.com

**Keywords:** serum, immunity, methodology, challenges, Israel

## Abstract

The Israel National Sera Bank (INSB) was established in 1997 in the Israel Center for Disease Control. The purpose of the INSB was to provide policymakers with data on the immunity status of the Israeli population against vaccine-preventable diseases, and on the extent and characteristics of exposure to emerging and re-emerging infectious diseases. The aim of this paper is to describe the methods, representativeness, and challenges in maintaining the INSB. The INSB comprises residual sera collected in six laboratories. By the end of 2019, 138,898 samples had been deposited in the INSB. These include samples from four community laboratories: 30.7% from the National Blood Service, 22.2% from Haifa and the Western Galilee, 21.7% from Soroka, and 0.7% from Jerusalem; and from two medical center laboratories: 18.6% from Schneider and 6.1% from Mayanei Hayeshua. The demographic characteristics of the sample at the end of 2019 closely resembled those of the general population. The main challenges addressed in maintaining the INSB relate to its representativeness, the possibility of repeated donors, costs, stability of antibody levels after long-term storage, ethical aspects, and the data available for each sample. The INSB is a unique, powerful, and necessary tool for assessing population immunity levels, based on serum samples collected over a long period of time.

## 1. Introduction

The Israel Center for Disease Control (ICDC) was established in 1994 as part of the Israeli Ministry of Health. The main purpose of the ICDC has been to identify information gaps and to provide policymakers with the data necessary for informed decision-making. 

In Israel, routine vaccines are provided for babies and children at Tipat Halav family care centers and at schools as part of the routine vaccination program and are free of charge [1]. Serological surveys have been shown to potentially entail the most direct and informative means of inferring the dynamics of a population’s susceptibility and level of immunity against vaccine-preventable and emerging infectious agents [2]. Thus, as part of its mission, the ICDC founded the Israel National Sera Bank (INSB) in 1997. The INSB is targeted at monitoring the immunity of the Israeli population against vaccine- and non-vaccine preventable disease, identifying population groups that are under-vaccinated, estimating time trends, and assessing immunization programs. The initial sampling scheme of the INSB followed the guidelines provided by the European Sero-Epidemiology Network (ESEN) [3]. The output of the INSB assists policymakers in evaluating immunization programs in Israel and in managing preventive measures against emerging and re-emerging infectious agents for which vaccines are not yet available.

In this paper, we aimed to describe the methodology, representativeness, and challenges in maintaining the Israeli National Sera Bank.

## 2. Materials and Methods

The INSB was designed as a collaboration between the Israel Ministry of Health, medical centers, healthcare funds’ community laboratories, the Medical Emergency Services in Israel (Magen David Adom) through their National Blood Service (NBS), and academia. 

Sample collection: Serum samples for the INSB are collected from six laboratories, all based on residual sera. As a division of Magen David Adom, located in Tel-Hashomer Medical Center in central Israel, the NBS provides the INSB with residual serum samples obtained from healthy blood donors. All the samples collected from the NBS are screened and test negative to human immunodeficiency virus 1/2 and are without active hepatitis B, hepatitis C, human T-lymphotropic virus, and syphilis infection. Healthcare fund community laboratories provide the INSB with residual serum samples obtained from individuals who perform routine or diagnostic blood tests. This source includes the following laboratories: the Haifa and Western Galilee laboratory of the Clalit Health Maintenance Organization (HMO) in northern Israel, Soroka laboratory of the Clalit HMO (non-hospitalized patients) in the south of Israel, and Jerusalem Clalit HMO in Central Israel. Two medical centers provide the INSB with residual sera of their patients: Schneider Children’s Medical Center in Petah-Tikva and Mayanei Hayeshua Medical Center in Bnei-Brak. At each site, an ICDC representative is recruited to collect samples according to predefined criteria. Table 1 describes, for each laboratory, the time the collection was established, the type of sample donors (healthy or patients), the number of samples collected every month, the type of laboratory (medical center or HMO), and the distribution of samples by age range, gender, population group (Jews and others vs. Arabs), and residential sociodemographic rank. The expected ratios represent the distribution of these characteristics in the total Israeli population.

Handling of the samples: Blood sample tubes are centrifuged around 2000–3000× *g*, according to the laboratories protocol. From each serum sample, a volume of 0.3–1.5 mL is collected, transferred to a 2.0 mL cryo tube, labeled with a unique internal identification number, and placed in a square 9 × 9 carton box. All sera are transported refrigerated to the ICDC and are stored in ultra-low temperature freezers (−80 °C) located at the Central Virology Laboratory facilities in the Sheba Medical Center campus. The position of the box within the freezer is carefully documented so that each sample can be easily accessed using the existing database. Samples are withdrawn from the database when the volume is drained. For each sample, the following data are saved: age, gender, residence (city), birth country, and population group (Jews and others, or Arabs). All the samples are kept anonymously. 

Quality control: Quality control measures are applied to verify the compatibility of each sample in the INSB to the prerequisites of each laboratory. Samples not meeting the prerequisites are withdrawn, but this number is lower than 10 every year. In addition, at the end of every collection year, the representativeness of the Sera Bank is evaluated, and adjustments are made as needed. For example, if a specific population group is found to be underrepresented, the prerequisites from the collection laboratories are adjusted. 

When the ICDC receives a project request, including details of age groups, gender, population group, and geographical distribution, the project is checked for applicability. Once approved, the required volume is transferred to the researcher, accompanied with an appropriate data file. A sample not containing the volume requested is replaced according to the researcher prerequisites. The samples are thawed, transferred to the project tubes, and re-frozen if the leftover sample volume is higher than 0.1 mL, or else disposed.

Ethics: The sera are collected within the regulatory capacity of the Israel Ministry of Health and approved by its legal department.

Statistical analysis: To describe the demographic characteristics of the samples included in the INSB, proportions were calculated by dividing the number within a specific population group (n) by the total population (N). To assess demographic representativeness, the distribution of the INSB population was compared to that of the Israeli population using data retrieved from the Israel Central Bureau of Statistics on the Israeli population in 2008 (the median of 1997–2019) [4]. We also divided the period into three according to collection patterns: 1997–2003, 2004–2010, and 2011–2019; and compared representativeness to the Israeli population in the median year of each period (2000 [4], 2007 [4], and 2015 [4], respectively). For each sample, sociodemographic rank was allocated based on the donor’s address using the socioeconomic residential classification published in 2008 by the Israeli Central Bureau of Statistics [5]. This rank is based on 14 variables and ranges between 1 (the lowest) and 10 (the highest). The data were analyzed using the SAS Enterprise Guide (version 7.12 HF5 (7.100.2.3472), SAS Institute Inc., Cary, NC, USA).

## 3. Results

**Figure 1:**
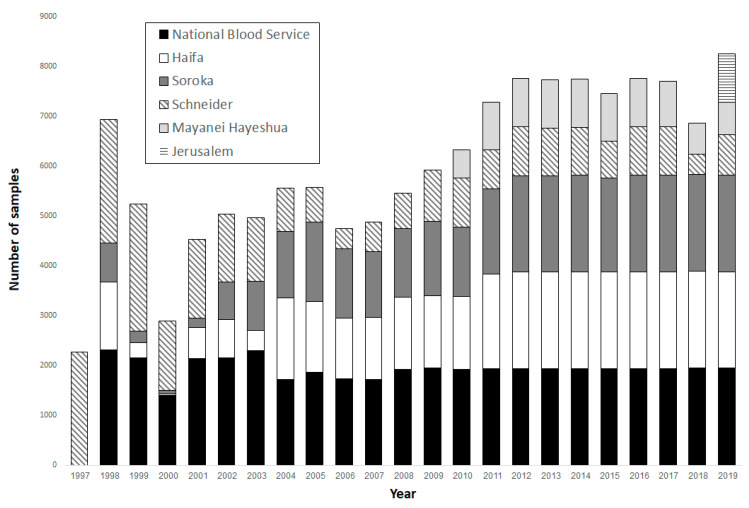
The distribution of Israel National Sera Bank samples collected as of December 2019, by collection site and year.

At the end of 2019, 138,898 samples had been deposited in the INSB. Between 1997 and 2012, the annual number of serum samples collected steadily increased. The annual number has since stabilized at around 7500 samples per year (Figure 1): 30.7% from the NBS, 22.2% from the Haifa and Western Galilee laboratory, 21.7% from the Soroka laboratory, 18.6% from the Schneider laboratory, 6.1% from the Mayanei Hayeshua laboratory, and 0.7% from the Jerusalem laboratory (Table 2). Distributions of age groups, gender, birth country, population group, and residential sociodemographic rank in each laboratory are presented in Table 2.

Compared to healthy blood donors, those who performed blood tests for routine or diagnostic purposes were older, of lower residential sociodemographic rank, and were more often females and Arabs (Table 3).

Table 4 compares demographic characteristics of the INSB population to those of the Israeli population at the median years of each period: between the period of 1997–2003 and the median year 2000, between 2004–2010 and 2007, and between 2011–2019 and 2015; and between the total collection period (1997–2019) and 2008. Significant differences were observed in the characteristics examined between the sera collected for the INSB and the Israeli population. These differences were less pronounced in the most recent period, 2011–2019. 

Inherent challenges faced by the INSB are representativeness, the possibility of repeated donors, annual costs of maintenance, long-term storage of sera, and ethical and legal aspects. Various means have been undertaken to mitigate these challenges. Below is a brief description of the challenges and means of mitigation.

Representativeness: The representativeness of the INSB is evaluated annually. The findings have prompted the introduction of new laboratories to bridge gaps when the existing laboratories did not have access to the underrepresented population groups. For example, in 2009 and 2018, two cities, Bnei-Brak and Jerusalem, with high rates of vaccine hesitance and refusal, were underrepresented in the INSB samples. In subsequent years, two laboratories in those cities were added to the INSB. Notably, we follow convenience sampling, mostly due to the very good cooperation with the Clalit HMO. This is the largest of the four national HMOs in Israel and represents 50% of the population [6]. 

Repeated donors: Both healthy blood donors and individuals performing blood tests for diagnostic or other purposes tend to donate and perform blood tests often. Thus, more than one sample may be archived from the same donor. Due to confidentiality, the samples are kept anonymously; thus, an analysis to identify repeated donors is inapplicable. However, the probability of collecting a sample from a specific donor of the hundreds or even thousands of samples collected every month is low; thus, the probability of a repeated donor is low. 

The annual cost of maintenance of the INSB is high and includes qualified personnel, equipment for long-term sample saving, and infrastructure. However, being a unique national resource, the ICDC covers these expenses through its annual budget. 

Long-term storage of sera at −80°: Previous studies have demonstrated that this does not affect many types of antibodies [7,8,9], in contrast to large differences in both directions that have been observed in serum levels of proteins, hormones, and lipids [10,11]. Nonetheless, studies investigating the stability of antibody levels over decades have not been performed, and information bias should be taken into account. 

Ethical and legal aspects: These relate to the privacy of the sample donors and are of high concern, as in other biobanks [12]. The National Sera Bank was approved by the legal department of the Israeli Ministry of Health and adheres to strict privacy rules; thus, this concern is redundant. The demographic data available for each sample are limited due to confidentiality and do not include important variables such as smoking and other behavioral habits, and health status.

## 4. Discussion

We described the methodology, characteristics, and main challenges of the INSB, a unique, national resource essential to monitoring population immunity. 

Several serum banks have been established around the world for various purposes. The Cancer Registry of Norway established the Janus Serum Bank Cohort in 1973, which consists of residual blood samples for cancer research [13,14]. The ESEN was established in 1996 to coordinate and harmonize serological surveillance of immunity to vaccine-preventable diseases in six European countries [3]. The ESEN2, established in 2001, included 22 European countries and aimed to standardize assay results and to enable comparisons of seroprevalence data across countries [15,16]. The EuroPrevall Serum Bank was an EU-funded multidisciplinary integrated project involving 16 European states, which started in 2005 and ended after 4 years. It was used to identify risk factors for food allergies [17]. The Denver Serum Bank was established in the 1950s and supported military research programs and other researchers nationally and internationally until the 1990s when a lack of funding and considerations of administration, space, and costs resulted in the destruction of all specimens [18]. The Netherlands established a national serum bank that aimed to evaluate the National Immunization Program [19], similar to the INSB. The U.S. Centers for Disease Control and Prevention presented a repository of serum samples collected for specific purposes [20]. Despite these initiatives, as has been suggested, a World Serology Bank, in contrast to national serum banks established locally, could be helpful in optimizing vaccination strategies and in the global task of eradicating vaccine-preventable infections such as polio, measles, and rubella [2]. 

We have shown an increase in the number of samples collected between 1997 and 2012, and stabilization since. The changes in the numbers of samples collected during this period are attributed to adjustments made due to annual quality control assessments.

We described two types of samples represented by the national serum bank, namely, of healthy blood donors and of individuals who performed blood tests for diagnostic or other purposes. However, we have shown that the samples from these two sources complement each other and together better represent the Israeli population, though not perfectly. The discrepancies observed in demographic characteristics between the National Sera Bank population and the Israeli population may be due to methodological differences in sample collection over the years. Since its establishment, laboratories were added to the INSB according to the representativeness (or lack thereof) of specific population groups. Currently, the sample collected does not fully represent the Israeli population, but the number of samples is planned to grow, and thus, a better representativeness is expected. Another explanation may be the deliberate oversampling of specific population groups that tend to avoid vaccinations. For example, the Jewish ultra-Orthodox population is often vaccine-hesitant, and due to its centrality in the incidence of infectious diseases, we made special efforts to sample this group. As we plan to further expand our sample in the near future, we expect the INSB to highly represent the Israeli population. In cases where a deviation is observed, we will try to amplify the number of samples from the under-represented subgroup in our laboratories, or to add a new laboratory which will meet the requirement.

The challenges associated with the maintenance and the use of the INSB, as detailed above, are mitigated and addressed through adaptations, adjustments, and continuous surveillance. 

A considerable number of projects have used the INSB, thus yielding a large number of papers published throughout the years [21,22,23,24,25,26,27,28,29,30,31,32,33,34,35,36,37]. Some of these have evaluated immunity of the population against vaccine-preventable diseases (diphtheria [21], Bordetella pertussis [22], influenza [23], hepatitis A [24], hepatitis B [25], measles [26], mumps [27], rubella [27,28], varicella [29], and polio [30]). Other projects have assessed exposure of the population to infectious agents, including emerging ones (herpes simplex viruses 1 and 2 [31], Helicobacter pylori [32], Toxoplasma gondii [33], hepatitis E [34], parvovirus [35], West Nile Fever Virus [36], MERS-CoV, Sindbis [37], and severe acute respiratory syndrome coronavirus 2 (SARS-CoV-2)). Further activities of the INSB are published on the Israel Center for Disease Control website (https://www.health.gov.il/English/MinistryUnits/ICDC/Units/labs/Pages/bank.aspx accessed on 25 February 2021). These data are highly important to public health. 

An example of the use of the INSB is our ability to assess trends of immunity against hepatitis A virus before and 12 years after the introduction of the vaccine using the same sample design [24]. In that study, we showed that the seropositivity rate among Jews and Arabs in age groups covered by the vaccine plan increased significantly after its introduction. Moreover, among Jews, the age groups not included in the vaccination program increased significantly [24]. Another study performed using the INSB demonstrated that infants between ages 6 and 11 months and children younger than 2 years showed the lowest seropositivity rates against measles [26]. These age groups had the highest attack rates of measles during the epidemic of 2018 in Israel [26]. 

## 5. Conclusions

The INSB is a powerful and necessary tool for the assessment of population immunity based on a well-defined collection plan, and on serum sample collection over more than two decades. The considerable challenges associated with its maintenance are continuously mitigated, thus ensuring the successful continuation of this unique national resource. The obstacles presented here may be useful for countries willing to establish a national serum bank, and the benefits of the information derived from the data deposit using the serum bank are highly important to public health.

## Figures and Tables

**Table 1:** Prerequisites of serum sample collection for the Israel National Sera Bank, by collection site.

Characteristic	National Blood Service	Haifa	Soroka	Schneider	Mayanei Hayeshua	Jerusalem
Date established	February 1998	March 1998	March 1998	June 1997	June 2010	January 2019
Type of donors (Healthy/Patients)	Healthy	Patients	Patients	Patients	Patients	Patients
Number of samples collected per month	162	162	162	81	81	81
Type of laboratory (Medical Center/HMO ^1^)	-	HMO	HMO	Medical Center	Medical Center	HMO
Age range	≥18 years	60 children aged <18 years 102 adults aged ≥18 years	60 children aged <18 years 102 adults aged ≥18 years	81 children aged <18 years	81 of all age groups	81 of all age groups
Gender ratio (Male: female)	1:1	1:1	1:1	1:1	1:1	1:1
Population group ratio (Jews and others: Arabs)	80%:20%	80%:20%	80%:20%	80%:20%	80%:20%	80%:20%

^1^ HMO = Health Maintenance Organization.

**Table 2:** Demographic characteristics of the individuals whose sera were collected for the Israel National Sera Bank, by collection site (1997–2019).

Variable	Category	Laboratory											
		NBS ^¥^		Haifa		Soroka		Schneider		MY ^£^		Jerusalem	
		*n*	%	*n*	%	*n*	%	*n*	%	*n*	%	*n*	%
Total		42,717	30.7	30,789	22.2	30,092	21.7	25,778	18.6	8552	6.1	970	0.7
Age group (years)	0–4	0	0.0	2217	7.2	3814	12.7	7098	27.6	407	4.8	68	7.0
	5–14	0	0.0	7835	25.4	5532	18.4	13,990	54.3	359	4.2	147	15.2
	15–19	4687	11.0	4627	15.0	3938	13.1	4663	18.1	361	4.2	75	7.7
	20–24	8656	20.3	685	2.2	835	2.8	9	0.0	997	11.7	56	5.8
	25–29	6704	15.7	677	2.2	673	2.2	6	0.0	713	8.3	57	5.9
	30–34	4865	11.4	660	2.1	666	2.2	2	0.0	663	7.8	57	5.9
	35–44	8062	18.9	1512	4.9	1483	4.9	2	0.0	879	10.3	93	9.6
	45–54	6270	14.7	1475	4.8	1026	3.4	0	0.0	553	6.5	68	7.0
	55–64	3169	7.4	3942	12.8	4080	13.6	0	0.0	784	9.2	76	7.8
	65–74	231	0.5	3861	12.5	4200	14.0	0	0.0	772	9.0	86	8.9
	75+	8	0.0	3298	10.7	3835	12.8	0	0.0	2064	24.1	187	19.3
Gender	Male	30,264	70.9	12,809	41.6	13,477	44.8	13,237	51.4	2939	34.4	497	51.2
	Female	12,417	29.1	17,978	58.4	16,608	55.2	12,523	48.6	5613	65.6	473	48.8
Birth country	Israel	33,832	79.3	22,846	74.2	19,484	65.0	21,970	87.5	5634	65.9	765	78.9
	Other	8810	20.7	7928	25.8	10,508	35.0	3137	12.5	2917	34.1	205	21.1
Population group	Jews and others	30,708	93.6	12,015	52.0	19,864	67.0	19,929	83.6	8002	99.9	165	97.1
	Arabs	2115	6.4	11,103	48.0	9808	33.0	3920	16.4	10	0.1	5	2.9
Sociodemographic rank	*n*; mean ± standard deviation	34,396; 5.7 ± 1.8		27,852; 4.8 ± 1.8		23,908; 4.0 ± 1.8		23,182; 5.4 ± 2.0		8404; 3.7 ± 1.7		917; 3.9 ± 0.7	

^¥^ NBS: National Blood Service. ^£^ MY: Mayanei Hayeshua.

**Table 3:** Demographic distributions of healthy blood donors and of individuals who performed blood tests for routine and diagnostic purposes.

Variable	Category	Healthy Blood Donators		Leftovers of Routine or Diagnostic Blood Samples		*p*-Value
		*n*	%	*n*	%	
Age group (years)	0–4	0	0.0	13,604	14.1	<0.01
	5–14	0	0.0	27,863	29.0	
	15–19	4687	11.0	13,664	14.2	
	20–24	8656	20.3	2573	2.7	
	25–29	6704	15.7	2120	2.2	
	30–34	4865	11.4	2046	2.1	
	35–44	8062	18.9	3967	4.1	
	45–54	6270	14.7	3122	3.2	
	55–64	3169	7.4	8882	9.2	
	65–74	231	0.5	8919	9.3	
	75+	8	0.0	9384	9.8	
Gender	Male	30,264	70.9	42,959	44.7	<0.01
	Female	12,417	29.1	53,195	55.3	
Birth country	Israel	33,832	79.3	70,699	74.1	<0.01
	Other	8810	20.7	24,695	25.9	
Population group	Jews and others	30,708	93.6	59,975	70.7	<0.01
	Arabs	2115	6.4	24,846	29.3	
Sociodemographic rank	*n*; mean ± Standard Deviation	34,396; 5.7 ± 1.8		84,263; 4.6 ± 1.9		<0.01

**Table 4:** Demographic characteristics of the individuals whose sera were obtained for the National Sera Bank, and the distribution of the Israeli population in 1997–2003, 2004–2010, and 2011–2019, and in the total period (1997–2019).

Variable	Category	1997–2003			2004–2010			2011–2019			1997–2019		
		INSB	2000 IP ^£^	*p*-Value	INSB	2007 IP ^£^	*p*-Value	INSB	2015 IP ^£^	*p*-Value	INSB	2008 IP ^£^	*p*-Value
Age group (years)	0–4	14.0	10.3	<0.01	7.8	10.2	<0.01	9.0	10.3	<0.01	9.8	10.2	<0.01
	5–14	30.3	18.3		21.3	18.2		14.7	17.9		20.1	18.2	
	15–19	15.6	8.7		13.8	8.1		11.8	7.8		13.2	8.1	
	20–24	8.2	8.5		6.5	7.9		8.9	7.3		8.1	7.8	
	25–29	7.3	7.9		5.8	7.6		6.2	7.0		6.4	7.5	
	30–34	4.8	6.6		4.4	7.4		5.4	6.9		5.0	7.4	
	35–44	8.4	12.0		6.4	11.9		10.1	12.8		8.7	12.0	
	45–54	7.4	11.2		5.1	10.5		7.4	10.0		6.8	10.3	
	55–64	3.1	6.6		11.2	8.4		9.9	9.0		8.7	8.7	
	65–74	0.6	5.5		9.1	5.2		8.0	6.1		6.6	5.1	
	75+	0.3	4.3		8.5	4.6		8.8	4.9		6.8	4.6	
Gender	Male	58.1	49.3	<0.01	54.5	49.4	<0.01	49.3	49.6	0.15	52.7	49.4	<0.01
	Female	41.9	50.7		45.5	50.6		50.7	50.4		47.3	50.6	
District	Jerusalem	4.2	11.9	<0.01	5.2	12.3	<0.01	4.9	12.5	<0.01	4.8	12.3	<0.01
	North	13.6	17.0		15.7	16.9		21.6	16.3		18.1	16.9	
	Haifa	17.7	12.9		19.4	12.1		13.1	11.6		15.9	12.0	
	Central	30.5	22.8		16.7	23.8		14.0	24.4		18.5	23.9	
	Tel Aviv	16.5	18.3		9.5	16.9		13.8	16.2		13.2	16.7	
	South	14.5	14.1		30.4	14.3		29.5	14.4		26.3	14.3	
	Judea and Samaria	3.2	3.0		3.2	3.7		3.3	4.5		3.2	3.9	
Birth country (among Jews)	Israel	90.2	61.8	<0.01	54.4	69.6	<0.01	70.3	75.6	<0.01	68.6	70.3	<0.01
	Other	9.8	38.2		45.6	30.4		29.7	24.4		31.4	29.7	
Population group	Jews and others	84.4	81.5	<0.01	83.0	80.1	<0.01	70.7	79.2	<0.01	77.1	79.9	<0.01
	Arabs	15.6	18.5		17.0	19.9		29.3	20.8		22.9	20.1	
Sociodemographic rank	*n*; Mean ± SD ^¥^	5.5 ± 1.9	5.3 ± 2.0	<0.01	5.2 ± 1.9	5.2 ± 1.9	<0.01	4.5 ± 2.0	5.2 ± 2.3	<0.01	4.9 ± 2.0	5.2 ± 1.9	<0.01

^£^ IP—Israel Population. ^¥^ SD—Standard Deviation.

## Data Availability

All relevant data are within the manuscript.
